# Evaluation of the New York City COVID-19 case investigation and contact tracing program: a cascade of care analysis

**DOI:** 10.1186/s12889-024-19838-3

**Published:** 2024-08-29

**Authors:** Sarah Conderino, Lorna E. Thorpe, Nadia Shilpi Islam, Carolyn A. Berry, Stefanie Bendik, Rachel Massar, Chuan Hong, Andrew Fair, Anna Bershteyn

**Affiliations:** Department of Population Health, New York University Grossman School of Medicine, New York, NY USA

**Keywords:** COVID-19, SARS-Cov-2, Case investigation, Contact tracing, Program evaluation, Exposure notification

## Abstract

**Background:**

New York City (NYC) was the first COVID-19 epicenter in the United States and home to one of the country’s largest contact tracing programs, NYC Test & Trace (T2). Understanding points of attrition along the stages of program implementation and follow-up can inform contact tracing efforts for future epidemics or pandemics. The objective of this study was to evaluate the completeness and timeliness of T2 case and contact notification and monitoring using a “cascade of care” approach.

**Methods:**

This cross-sectional study included all SARS-CoV-2 cases and contacts reported to T2 from May 31, 2020 to January 1, 2022. Attrition along the “cascade of care” was defined as: (1) attempted, (2) reached, (3) completed intake (main outcome), (4) eligible for monitoring, and (5) successfully monitored. Timeliness was assessed: (1) by median days from a case’s date of testing until their positive result was reported to T2, (2) from result until the case was notified by T2, and (3) from a case report of a contact until notification of the contact.

**Results:**

A total of 1.45 million cases and 1.38 million contacts were reported to T2 during this period. For cases, attrition occurred evenly across the first three cascade steps (~-12%) and did not change substantially until the Omicron wave in December 2021. During the Omicron wave, the proportion of cases attempted dropped precipitously. For contacts, the largest attrition occurred between attempting and reaching (-27%), and attrition rose with each COVID-19 wave as contact volumes increased. Attempts to reach contacts discontinued entirely during the Omicron wave. Overall, 67% of cases and 49% of contacts completed intake interviews (79% and 57% prior to Omicron). T2 was timely, with a median of 1 day to receive lab results, 2 days to notify cases, and < 1 day to notify contacts.

**Conclusions:**

T2 provided a large volume of NYC residents with timely notification and monitoring. Engagement in the program was lower for contacts than cases, with the largest gap coming from inability to reach individuals during call attempts. To strengthen future test-and-trace efforts, strategies are needed to encourage acceptance of local contact tracer outreach attempts.

**Supplementary Information:**

The online version contains supplementary material available at 10.1186/s12889-024-19838-3.

**Table:** 

**Text box 1. Contributions to the literature**	
• Achieving complete and timely case investigation and contact tracing was a priority of the SARS-CoV-2 pandemic.	
• Our evaluation of the New York City COVID-19 case investigation and contact tracing program found that a large volume of NYC residents were provided with timely notification and monitoring. However, engagement in the program was lower for contacts than cases, with the largest gap coming from inability to reach individuals during call attempts.	
• These results suggest that the program was largely successful at timely notification and monitoring of individuals with COVID-19 but additional strategies may be needed to improve engagement with contact tracing efforts.	

## Background

Contact tracing is a key function of public health practice. Timely notification of positive cases and identification of exposed contacts can help to reduce the spread of an infectious disease when individuals need to isolate or quarantine to interrupt person-to-person transmission. Contact tracing was a priority at the start of the SARS-CoV-2 pandemic due to the lack of pharmaceutical control measures, evidence-based treatment protocols, and widespread testing [1]. 

New York City (NYC) was the first US epicenter of transmission, with more than 30,000 cases identified by March 29, 2020 [2]. Within three months of the first case identified, NYC Health + Hospitals (H + H, NYC’s public hospital system), in partnership with the NYC Department of Health and Mental Hygiene (DOHMH), other city agencies, and a large network of community partners, launched the NYC Test & Trace (T2) COVID-19 contact tracing program [3]. T2 had three key objectives: to identify and isolate cases (termed the “Test” pillar); to reduce transmission through contact tracing (“Trace”); and to provide resources to residents in need of support during their isolation or quarantine periods (“Take Care”).

The “Trace” pillar of T2 was designed to provide timely notification of positive SARS-CoV-2 test results or exposure to potentially-infectious individuals, as well as monitoring of self-isolation or quarantine and identification and further tracing of close contacts of all New Yorkers who tested positive for SARS-CoV-2. This process involves multiple steps, with potential attrition from the tracing process at each step. The “cascade of care” approach, popularized in the global fight against HIV/AIDS, visualizes attrition in a healthcare process in a stepwise series of drop-offs, showing how each stage of attrition affects the number of people who ultimately achieve a desired outcome [4–6]. Applying a cascade of care analysis to contact tracing efforts allows for the comparison of the number of individuals lost at each point in the process and their multiplicative effect on the number of people who successfully received the service of interest. This approach can help to evaluate performance and prioritize areas for improvement in a complex, multi-stage healthcare process.

As part of a larger, mixed-methods external evaluation conducted by a research team at NYU Grossman School of Medicine, the goal of this study was to assess points of attrition and timeliness of the T2 Trace pillar, including the completeness and timeliness of case notification and contact tracing. This work extends prior evaluations of T2 by applying a cascade of care approach to investigate key points of attrition and cumulative losses along the stages of contact tracing [7, 8]. This analysis serves as both a retrospective evaluation and a basis for recommendations to improve contact tracing efforts for future epidemics and pandemics.

## Methods

### Study population

This analysis included all SARS-CoV-2 cases and exposed contacts residing in NYC and eligible for T2, excluding those identified from congregate settings (e.g., nursing homes) who were managed outside of T2. Because individuals could become re-infected or re-exposed throughout the pandemic, analysis was performed at the event-level (e.g., each SARS-CoV-2 infection or new exposure to SARS-CoV-2) rather than the individual-level.

Cases were de-duplicated if they occurred in the same individual within the same year before June 9, 2021, or within a 90-day period after June 9, 2021 (once evidence of re-infection was established). Cases included both confirmed positive and presumed positive. Confirmed cases included a new positive PCR or antigen test. Presumed positive disease events included instances when individuals were reported as a close contact of a confirmed positive case and met the clinical criteria for COVID-19 without evidence of a positive PCR or antigen test (Appendix).

Exposed contacts were de-duplicated during contact investigators’ workflows based on name, address, and phone number, but the time period for de-duplication was not known. Contacts identified through case interviews were considered to be exposed when within ≤ 6 feet of a case for a total of ≥ 15 min over a 24-hour period during the case’s infectious period. Cases were considered infectious two days before symptom onset for symptomatic cases, or two days before positive molecular or antigen test for asymptomatic cases, until 10 days after symptom onset or positive test (14 days prior to October 1, 2020).

While the majority of intake was completed by phone, some was conducted in-person. Data did not allow for the disaggregation of intake modality, but monitoring modality was collected (telephone calls vs. SMS messaging). The duration and mode of monitoring over which individual cases and contacts were assessed for outcomes changed over the course of this evaluation. The duration of monitoring was initially 14 days and was shortened to 10 days in December 2020. The modality of monitoring was initially telephone calls and was supplemented with automated SMS messaging in fall 2020.

### Definition of tracing cascades and timeliness

Tracing cascade and timeliness metrics were defined in collaboration with H + H in order to align with indicators reported in real-time on a public dashboard maintained by T2 [9]. Separate tracing cascades were developed for cases and contacts. Each included five steps: (1) “attempted” – staff made at least one notification attempt,  (2) “reached “– staff successfully reached the individual,  (3) “completed intake” – the individual completed the intake interview (main outcome measure), (4) “eligible for monitoring” – the individual completed intake before the end of the monitoring period, and (5) “successfully monitored” – the individual completed at least one monitoring interaction when ≤ 3 monitoring days remained at intake or at least two monitoring interactions when ≥ 4 monitoring days remained at intake. Cases and contacts with incomplete information (e.g., phone number or address) were routed to different investigation queues and were not attempted by case or contact notification staff. For individuals who provided information of at least one contact, a secondary case investigation cascade metric was constructed (termed “provided contacts”). More detailed definitions for each metric are provided in the Appendix.

Timeliness of the case investigation cascade was defined as median days from the case’s specimen collection until first notification attempt by T2. We further sub-divided this into lab timeliness (from specimen collection until upload of the positive case’s data into the T2 system) and notification attempt timeliness (from upload of the positive case’s data until first notification attempt by T2). Timeliness of the contact intake cascade was defined as the median number of days from contact elicitation to first contact notification attempt.

### Data analysis

The H + H Data, Analytics, and Product Team extracted and aggregated T2 case notification and contact tracing data from the operational Salesforce system in strata of week, ZIP Code Tabulation Area (ZCTA), age group (0–17, 18–44, 45–64, 65+, or Unknown), gender (Women; Men; Other; Unknown), and race/ethnicity (Black; White; Other; Asian; Unknown). Data were inclusive of all cases and exposed contacts referred to T2 from May 31, 2020 to January 1, 2022.

For each tracing cascade metric, we calculated the proportion of the total cases and contacts that reached a given cascade step (using total cases or contacts as the denominator) and the attrition, or percent difference, from the prior step in the cascade (using the number of cases or contacts reaching the prior step as the denominator). We examined trends and patterns in the tracing cascades and timeliness by week, variant wave, demographics, and neighborhood. Unlike other studies that defined variant waves based on sequenced specimens, [10, 11] this evaluation defined variant waves for periods where the NYC case count exceeded 1,000 cases per day to assess the impact of changing case volume, rather than viral genotype, on the tracing program. Waves were defined as: Alpha = 11/8/20–5/1/21; Delta = 7/25/21–10/9/21; Omicron = 11/14/21–1/1/22; all else = between-waves [12]. In secondary analysis, we compared cascades by recommended monitoring duration (14 days: 5/31/20–12/5/20, 10 days: 12/6/20–1/1/22) and monitoring modality (telephone: 5/31/20–8/1/20, telephone/SMS: 8/2/20–1/1/22).

## Results

**Fig. 1:**
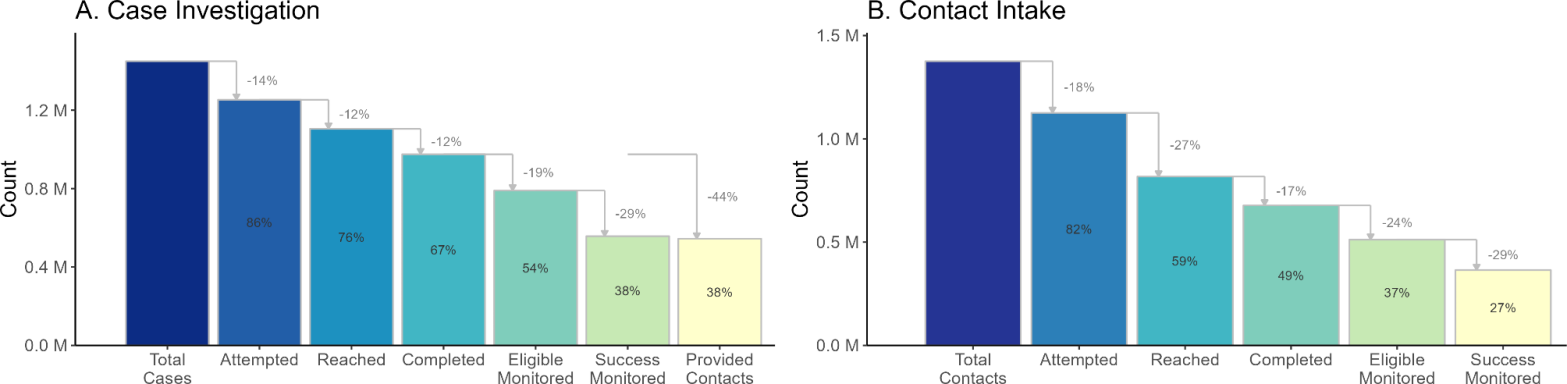
NYC T2 case investigation and contact intake cascades, May 31, 2020 – January 1, 2022. * Data Source: New York City Test & Trace (T2) case notification and contact tracing data. (https://med.nyu.edu/departments-institutes/population-health/divisions-sections-centers/epidemiology/sites/default/files/cimph-test-and-trace-report.pdf). **Panel A**: Case investigation cascade; **Panel B**: Contact intake cascade. Percentage labels outside the bars represent the percent decrease from the prior group displayed by the arrow; percentages inside the bars represent the percent of total cases that reached the cascade category

From May 31, 2020 to January 1, 2022, 1,449,844 cases and 1,375,455 contacts were reported to T2. Overall during the study period, attrition occurred evenly across the first three steps of the case investigation cascade, with approximately 12% loss at each step of attempting, reaching, and completing (Fig. 1A). More substantial attrition occurred after intake, with 19% ineligible for monitoring due to late completion of intake and 29% of those eligible not successfully monitored. Contacts were reported by 56% of cases who completed intake. Among cases who provided at least one contact, there was a mean of 2.4 contacts reported per case. Overall, 49% of contacts completed tracing, with the largest absolute attrition between those attempted and those reached (-27%, Fig. 1B). Large attrition also occurred after intake, with 24% of contacts ineligible for monitoring due to late completion of intake and 29% of those eligible not successfully monitored.

**Fig. 2:**
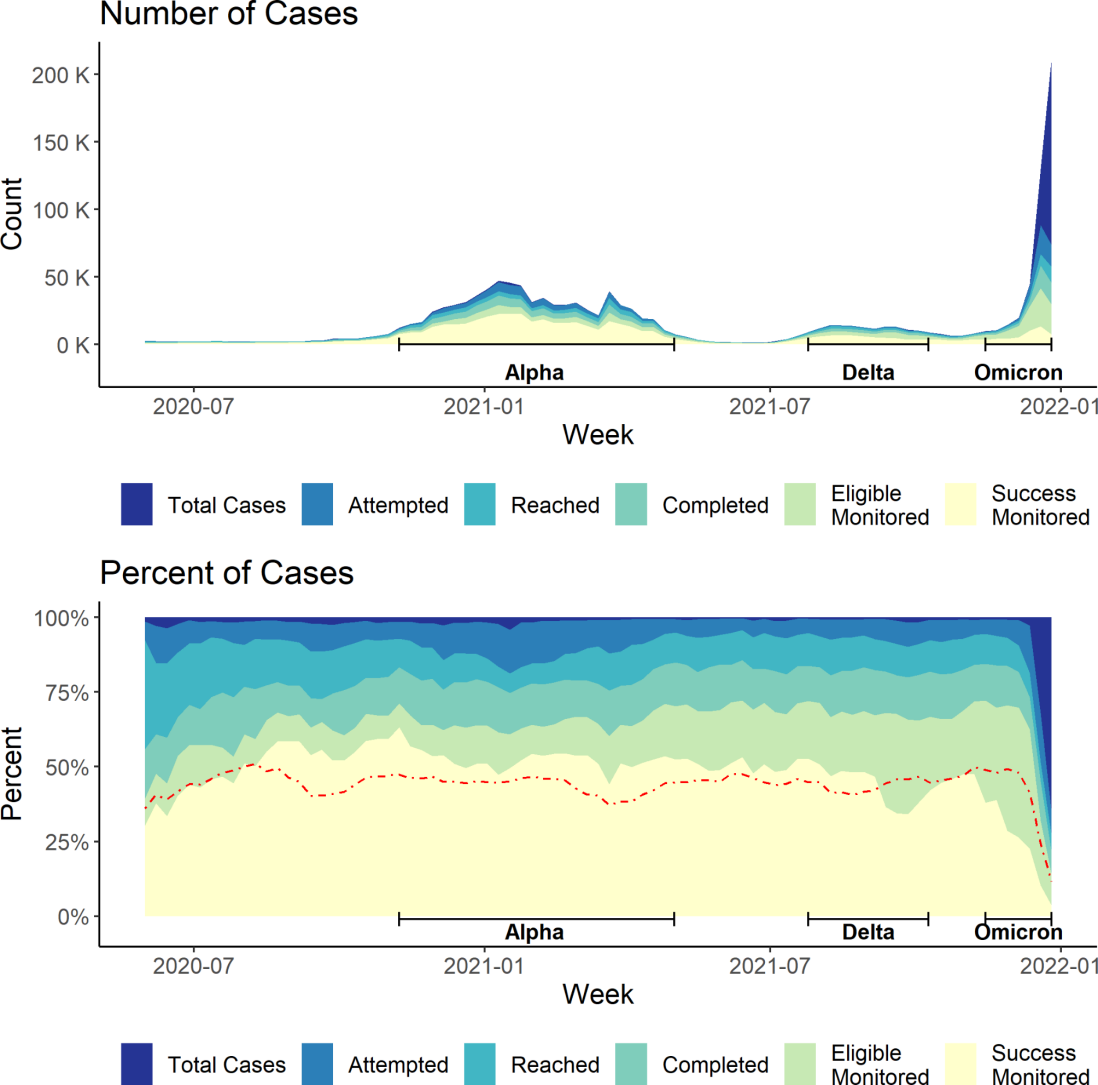
NYC T2 case investigation cascade by week, May 31, 2020 – January 1, 2022. * Data Source: New York City Test & Trace (T2) case notification and contact tracing data. (https://med.nyu.edu/departments-institutes/population-health/divisions-sections-centers/epidemiology/sites/default/files/cimph-test-and-trace-report.pdf). Red dashed line represents the percentage of total cases who provided contacts. Waves were defined as: Alpha = 11/8/20–5/1/21; Delta = 7/25/21–10/9/21; Omicron = 11/14/21–1/1/22

Prior to the Omicron wave, attrition in the first three steps of the case investigation cascade did not change substantially over time (Fig. 2). Modest gains were observed in completion of intake in the program’s early weeks, with case completion rates rising steadily from 56% at program launch to above 75% within the first three months. Pre-Omicron, 99% of cases were attempted, 89% reached, and 79% completed intake. During the Omicron wave, the proportion of cases attempted dropped precipitously (-41%, Fig. 2), reflecting a surge in cases that exceeded the program’s capacity, with over 400,000 cases reported within the seven weeks of the Omicron wave compared to approximately 1 million cases in the preceding 1.5 years. Subsequent attrition in reached (-19%) and completed intake (-14%) were comparable to earlier waves and in between-wave periods. Overall, 59% of cases were attempted, 48% reached, and 41% completed intake during Omicron.

Later steps in the case investigation cascade, occurring after completed intake, were more sensitive to changes over time. During the Alpha and between-wave periods, approximately 80% of eligible cases were successfully monitored. Successful monitoring began to fall during the Delta wave, when 65% of eligible cases were successfully monitored, and declined further during the Omicron wave, when only 35% of eligible cases were successfully monitored. Attrition in cases providing contacts increased modestly at program launch, from approximately 30% of cases not providing contact information in the first two months of the program to approximately 40% by September 2020.

**Fig. 3:**
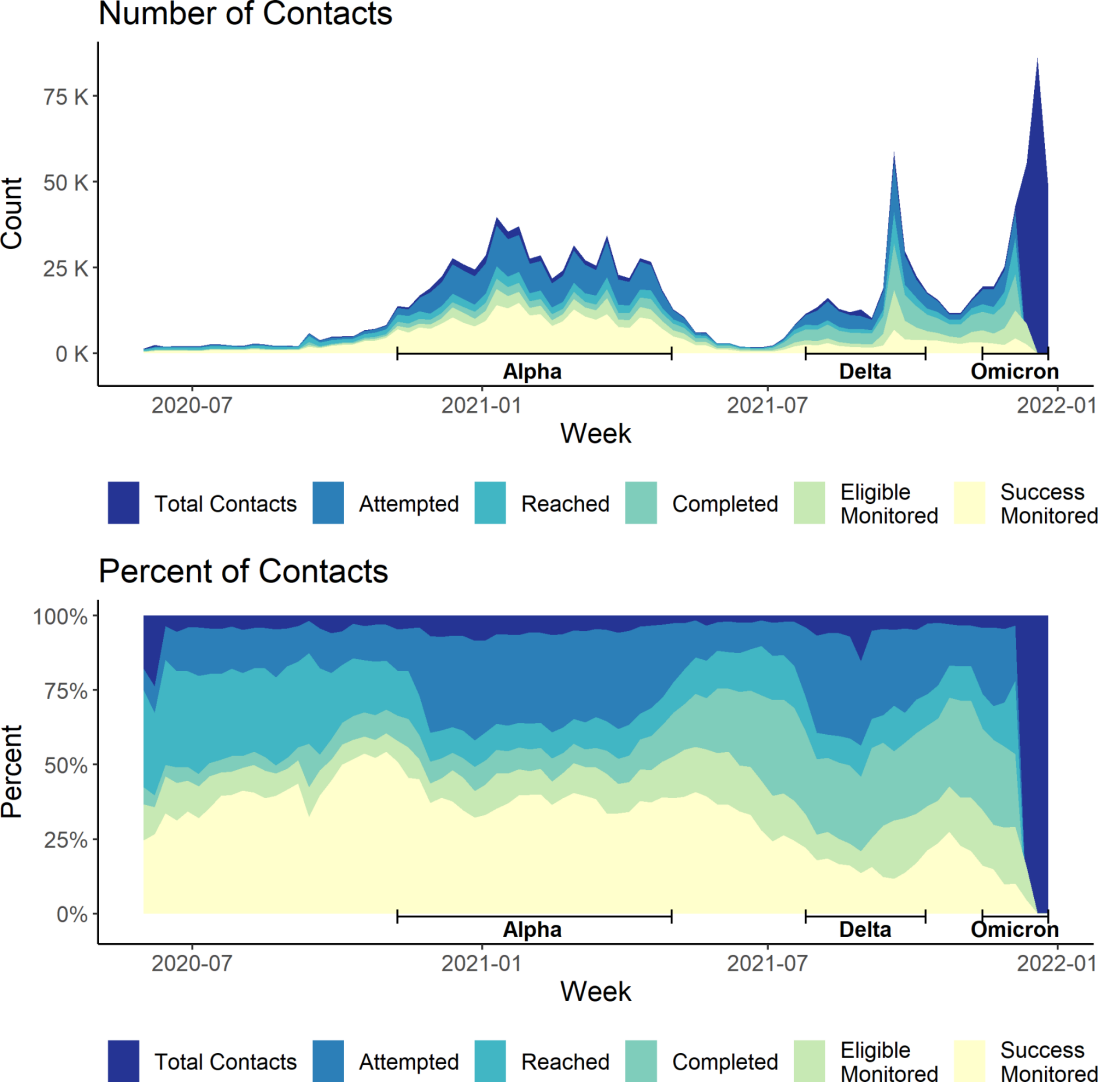
NYC T2 contact intake cascade by week, May 31, 2020 – January 1, 2022. * Data Source: New York City Test & Trace (T2) case notification and contact tracing data. (https://med.nyu.edu/departments-institutes/population-health/divisions-sections-centers/epidemiology/sites/default/files/cimph-test-and-trace-report.pdf). Waves were defined as: Alpha = 11/8/20–5/1/21; Delta = 7/25/21–10/9/21; Omicron = 11/14/21–1/1/22

The contact intake cascade was more sensitive to surges in infections over time than the case notification cascade (Fig. 3). While attempts were made to reach almost all contacts during the Alpha and Delta waves (94%), attrition from attempted to reached was twice as high as lower volume between-wave periods (-30% vs. -15%). In contrast, the Omicron wave was characterized by a substantial attrition in notification attempts, with only 35% of total contacts attempted during this period. There was also more attrition of reached contacts not completing tracing during the Omicron wave (-24%) compared to the Alpha (-15%) and Delta (-17%) waves. As with the case tracing cascade, the proportion of contacts who were successfully monitored declined during the Delta wave, when 51% of eligible contacts were successfully monitored. This declined further during the Omicron wave, where only 37% of eligible contacts were successfully monitored.

Case investigation cascades differed modestly by age (Appendix), with greater notification attempts among cases aged 65 years or older (92% vs. 85% for those aged 18–44 years). However, the proportion of cases with completed intake was relatively consistent across demographic groups (~ 68%). Case race/ethnicity and contact demographics could not be ascertained reliably due to large amounts of missing data (> 30%). Case investigation and contact intake cascades also did not differ substantially by neighborhood, with a median completion rate by neighborhood of 68% for cases and 71% for contacts (Appendix).

**Fig. 4:**
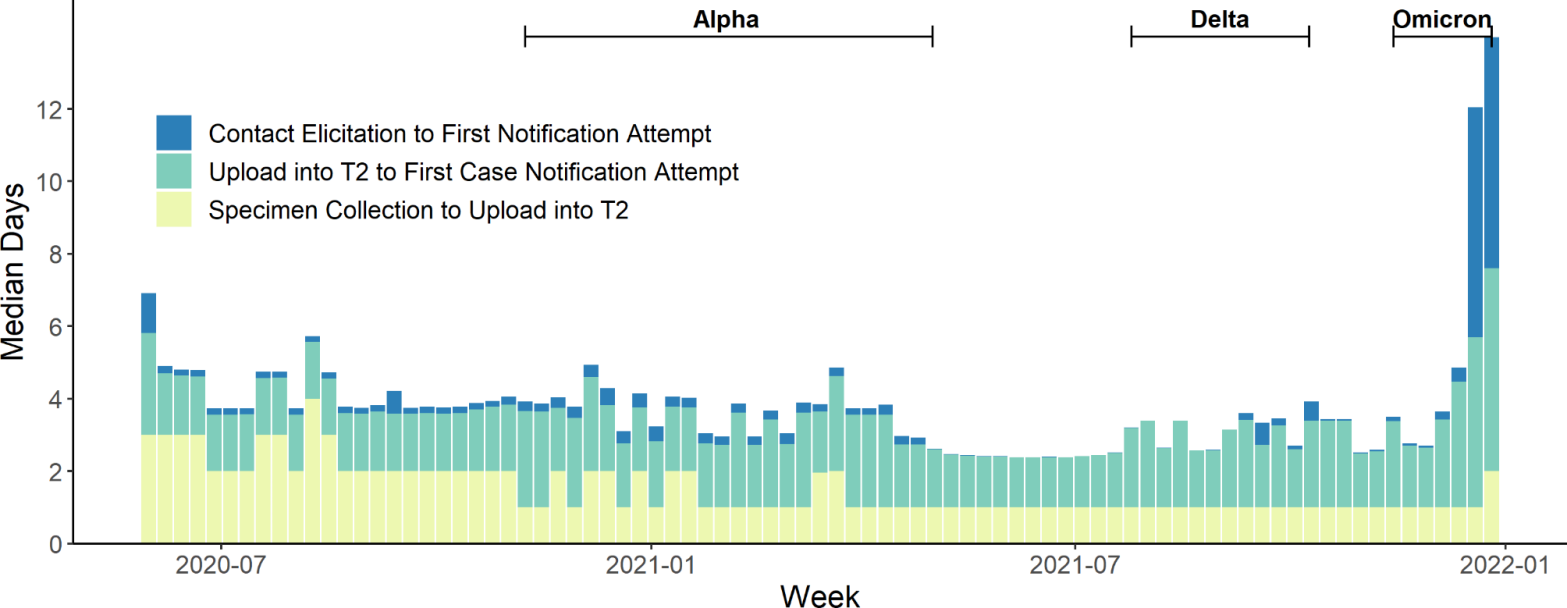
Timeliness of NYC T2 case and contact notification by week, May 31, 2020 – January 1, 2022. * Data Source: New York City Test & Trace (T2) case notification and contact tracing data. (https://med.nyu.edu/departments-institutes/population-health/divisions-sections-centers/epidemiology/sites/default/files/cimph-test-and-trace-report.pdf). Waves were defined as: Alpha = 11/8/20–5/1/21; Delta = 7/25/21–10/9/21; Omicron = 11/14/21–1/1/22

Overall, notification of case or contact status was timely, with a median of one day for lab results, two days for case notification, and 0.24 days for contact notification. After initial lab delays at the start of the pandemic response, timeliness of lab results was relatively stable (Fig. 4). Timeliness of case notification and contact notification worsened during the Omicron wave, where the median time from case upload into the T2 system to first notification attempt was four days and median time from contact elicitation to first notification attempt reached over six days.

Secondary analyses demonstrated that the proportion of cases and contacts opting into text monitoring was relatively stable at approximately 60% over time, until the last two weeks of December 2021, when approximately 80% of cases and contacts were monitored via text. In addition, while the proportion of cases eligible for monitoring was stable (~ 80%) pre- and post-December 2020, when the duration of monitoring decreased from 14 to 10 days, the proportion of contacts eligible for monitoring decreased from 87 to 74% due to not completing intake within the monitoring period. This decrease in the proportion of contacts eligible for monitoring persisted even when the Omicron wave was excluded from the analysis.

## Discussion

The T2 program provided relatively comprehensive and timely notification of positive test results, prior to overwhelmingly high case volumes during the Omicron wave. Rates of case completion were stable by demographics and neighborhood of residence. These findings were consistent with T2 outcomes published by practitioners at NYC H + H and NYC DOHMH [7, 8]. However, engagement of contacts was substantially more challenging, with almost half reporting having no exposed contacts and over one-quarter of contacts unable to be reached. Initial case contact notification attempts were timely, but secondary analyses demonstrated that almost one-quarter of contacts completed intake outside of the 10-day monitoring period. An additional one-third of cases and contacts who were eligible were not successfully monitored. Case and contact notification attempts dropped precipitously during the Omicron wave.

These findings suggest that T2 was generally successful at rapidly scaling up to provide complete and timely case investigation to a very large and highly diverse urban population. Assuming cases complied with isolation guidelines after being notified of their COVID-19 infection, these successes may have contributed to reductions in disease transmission [13]. While these findings aligned with observed high levels of successful case interviews in other urban contact tracing programs, such as those in San Francisco (85%) and King County, Washington (82%), T2 served a much larger number of “clients” [14, 15]. T2 case completion was also considerably higher than rates observed in Chicago (37%) [16]. Other published papers have described strategies employed by T2 that may have contributed to high case completion rates and the wide, including contracting with vendors to quickly hire large numbers of contact tracing and community engagement/outreach staff and partnering with telephonic translation services to meet diverse language needs of NYC [7, 8, 17, 18]. Further, in response to staff feedback and increasing case volume, refinements were made in tracing protocols to shorten call scripts and transition to text messaging modalities. T2’s door-to-door outreach may also have been a successful supplemental strategy that improved completion rates.

Our findings regarding the contacts’ cascade suggest that a major challenge for T2 was soliciting accurate information for exposed contacts, limiting its effectiveness in reducing pre-symptomatic or asymptomatic spread of COVID-19 [19]. This challenge was not unique to NYC, with contact tracing programs from across the U.S. reporting low rates of contact elicitation or difficulties reaching named contacts [15, 16, 20–26]. Integration of digital contact tracing technologies, such as voluntary smartphone applications that use geolocation tracking to notify users when they have been in proximity to an individual who had COVID-19, could help improve the completeness and timeliness of contact notification [27, 28]. However, these types of solutions may exacerbate inequities among those who do not participate [27, 28]. Digital contact tracing could supplement but not replace the need for traditional contact tracing programs.

Declines in the proportion of cases reporting contacts may reflect fatigue or changes in public attitude over the duration of the pandemic. Studies have observed changes in public attitude towards the COVID-19 pandemic associated with changes in official guidance, the spread of disinformation and misinformation, and historical inequity-driven medical mistrust [29, 30]. Public understanding and trust in contact tracing programs is critical to encourage participation [31]. Despite extensive community engagement activities to legitimatize T2 notification calls, qualitative interviews with T2 staff and cases served by the program have cited lack of trust as a key barrier to providing information for contact tracing efforts [17]. Multifaceted strategies, including educational campaigns to address misinformation, media campaigns to educate the public about the purpose of contact tracing, and staff trainings on building rapport with callers should be explored to strengthen future tracing efforts [32, 33]. Increased uptake of vaccines may have also contributed to the decline in contact tracing efforts, with almost 50% of NYC residents receiving one dose by May 2021 and almost 80% receiving one dose by January 2022 [34]. 

Importantly, the significant surge in cases and contacts during the Omicron wave appears to have rapidly exceeded the program’s capacity for universal contact tracing. In response to this surge, T2 moved quickly to deprioritize universal tracing as a primary mitigation strategy, successfully transitioning to fully passive SMS text notification on December 20, 2021 for contacts and January 8, 2022 for cases. Other strategies, such as transitioning to targeted tracing efforts in order to prioritize essential workers or vulnerable populations, could ensure that resources are available for those in high-risk jobs or communities during periods of high case surge [35]. The use of alternative notification or monitoring modalities can also potentially improve the timeliness of tracing efforts. Passive notification could protect against staff and “client” burnout, as many call recipients may become overwhelmed by daily monitoring calls during periods of isolation or quarantine [17]. The observed low success of monitoring likely reflects recipients screening calls or opting not to respond from such burnout.

This evaluation was limited to using aggregate data on pre-specified metrics that were defined in collaboration with NYC H + H. As such, we could not conduct sensitivity analyses on metric definitions or further explore timeliness and attrition during monitoring efforts. For example, we could not distinguish whether the sizable proportion of contacts who were ineligible for monitoring was due to delays before or after the individual was successfully notified of their exposure status. A deeper understanding of when cases and contacts were successfully notified of their infection or exposure status, rather than when first notification attempts were made, could facilitate future efforts to examine the extent to which T2 activities mitigated the spread of COVID-19. In addition, the completeness of contact demographics and race/ethnicity among cases was poor, which limited our ability to assess equity. Robust collection of other risk factors or social determinants of health would improve assessment of equitable reach and inform operations of future contact tracing efforts. For most of the study period, cases were highly representative of NYC residents with a laboratory confirmed positive PCR or antigen test, or close contacts presumed to be positive based on symptoms. Representativeness was likely lower during the first weeks of program launch, when testing capacity was still limited, and during late-2021 when at-home testing practices increased [17]. These data also did not include detailed client outcomes or vaccination status, which may have affected responsiveness to contact tracing efforts.

## Conclusions

The NYC T2 program succeeded at providing millions of NYC residents with timely notification and monitoring prior to the Omicron surge, providing an important blueprint for future contact tracing efforts. However, it was less successful at reaching exposed contacts, suggesting that new strategies are needed to improve acceptance of outreach attempts by local contact tracers. The use of text messaging, instead of daily calls, may facilitate notification and monitoring efforts during high-surge periods or in contact tracing efforts more broadly. Further, targeted rather than universal contact tracing may be preferable during pandemic periods of high case surge, especially when asymptomatic transmission is occurring. Future studies should thoroughly evaluate the extent to which universal contact tracing mitigated the disease’s spread and the cost-effectiveness of these efforts.

### Electronic supplementary material

Below is the link to the electronic supplementary material.

Supplementary Material 1

## Data Availability

The data that support the findings of this study are available from the New York City Health and Hospitals Corporation but restrictions apply to the availability of these data, which were used under license for the current study, and so are not publicly available. Data are however available from the authors upon reasonable request and with permission of the New York City Health and Hospitals Corporation. Please contact Sarah Conderino (sarah.conderino@nyulangone.org) for more information.

## Abbreviations

NYC: New York City
ZCTA: Zip Code Tabulation Area
T2: Test & Trace
DOHMH: Department of Health and Mental Hygiene
H + H: NYC Health + Hospitals
