# Supplementary material for: Evaluation of the New York City COVID-19 case investigation and contact tracing program: a cascade of care analysis
Source: BMC Public Health. 2024 Aug 29;24:2356. doi: 10.1186/s12889-024-19838-3 (PMC11363647; doi:10.1186/s12889-024-19838-3)
Supplement: Supplementary file 1 — Supplementary Material 1 [file 12889_2024_19838_MOESM1_ESM.docx]

**Appendix**

**Table of Contents**

eTable 1. Case Investigation Cascade Definitions……………………………………………………………………..2

eTable 2. Contact Intake Cascade Definitions…………………………………………………………………………5

eFigure 1. Case Investigation Cascade by Age Group, May 31 2020–January 1, 2022……………………………….7

eFigure 2. Case Investigation Cascade by Gender, May 31 2020-January 1, 2022……………………………………7

eFigure 3. Percent of Cases (Panel A) and Contacts (Panel B) with Completed Intake by ZCTA, May 31 2020-……8 January 1, 2022

eTable 3: Numbers and Proportions of Clients Along the Case Investigation Cascade……………………………….9

eTable 4: Numbers and Proportions of Clients Along the Contact Intake Cascade…………………………………...9

eTable 1. Case Investigation Cascade Definitions

| **Metrics** | **Definition for cases** |
| --- | --- |
| Confirmed Positive Cases | Total number of disease events with positive PCR or antigen test in Trace database. All positive molecular and antigen tests were reported to the Department of Health and Mental Hygiene (DOHMH) and then added to the Trace database.  Cases that were identified from congregate settings, were residents of other jurisdictions, were identified due to death records without an antigen result, or were identified too late/outside of the period of diagnosis and would no longer have been eligible for follow up were not included as cases in the T2 data.  Confirmed positive cases were de-duplicated following DOHMH guidelines - if the person had positive COVID-19 results with collection dates at least one year (before June 9, 2021) or at least 90 days (after June 9, 2021) apart, then the data would include multiple disease events for the same person. Confirmed positive cases were also de-duplicated during case investigator workflows based on first/last name, address, and phone number, but the time period for deduplication was not known. |
| Presumed Positive Cases | Total number of events of individuals who were reported as a close contact of confirmed positive case and who met the clinical criteria for COVID, but who did not have laboratory evidence of positivity (including a molecular or antigen test). From the start of Trace until April 2021, any contact with one symptom was queued for case investigation; after April 2021, only contacts meeting CSTE criteria were queued.  Following Council of State and Territorial Epidemiologists (CSTE) criteria, cases are presumed positive if they meet the following:   - Two of the following symptoms: Fever, chills, muscle aches, headache, sore throat, vomiting, diarrhea, fatigue, congestion, OR - One of the following symptoms: Cough, shortness of breath, new loss of smell or taste   Presumed positive cases were de-duplicated during case investigator workflows based on first/last name, address, and phone number, but the time period for deduplication was not known. |
| Total Cases | Sum of confirmed and presumed positive cases reported to T2. |
| Attempted | Individuals for which the T2 tracer made at least 1 telephone attempt, including call dispositions of “busy/no answer”, “incorrect or inactive number”, “left voicemail”, “three failed attempts”, “unable to locate – final”, “call back requested”, “call dropped”, “call back scheduled”, “duplicate/already completed”, “emergency”, “in progress”, “language barrier”, “refused, call completed”, “incapable of responding/no proxy”, “out of jurisdiction”, “potentially deceased”, “referred to NYC Health Department Congregate Settings Team”, “refused – reports vaccination”, and “vaccine immune”.  The proportion of cases attempted was calculated at the number attempted divided by the total cases. Attrition from total cases to attempted was calculated as the percent difference from total cases to cases attempted. |
| Reached | Cases that were reached were defined as those whom the T2 tracer reached, with any call disposition of “call back requested”, “call dropped”, “call back scheduled”, “duplicate/already completed”, “emergency”, “in progress”, “language barrier”, “refused, call completed”, “incapable of responding/no proxy”, “out of jurisdiction”, “potentially deceased”, “referred to NYC Health Department Congregate Settings Team”, “refused – reports vaccination”, and “vaccine immune”.  The proportion of cases reached was calculated at the number reached divided by the total cases. Attrition from attempts to reached was calculated as the percent difference from cases attempted to cases reached. |
| Completed | Cases that were completed were defined as those who completed the intake interview, with call dispositions “call completed,” “incapable of responding/no proxy,” “out of jurisdiction,” “potentially deceased,” and “referred to NYC DOHMH Congregate Settings Team”.  The proportion of cases completed was calculated at the number completed divided by the total cases. Attrition from reached to completed was calculated as the percent difference from cases reached to cases completed. |
| Eligible monitored | Cases that were eligible for monitoring were defined as those with completed intake and >0 days of monitoring left. The monitoring period was originally specified as 14 days but was changed to 10 days in December 2020.  The proportion of cases eligible for monitoring was calculated at the number eligible divided by the total cases. Attrition from completed to eligible was calculated as the percent difference from completed to eligible for monitoring. |
| Success monitored | Cases that were successfully monitored were defined as those with either at least 1 complete monitoring interaction with ≤3 or monitoring days or at least 2 complete monitoring interactions with >4 monitoring days.  The proportion of cases successfully monitored was calculated as the number successfully monitored divided by the total eligible for monitoring. Attrition from eligible to successful monitoring was calculated as the percent difference from eligible to successfully monitored. |
| Provided contacts | Cases that provided contacts were defined as those who provided name and contact information for at least 1 contact.  The proportion of cases who provided contacts was calculated as the number provided contacts divided by the total cases. Attrition from completed to provided contacts was calculated as the percent difference from completed to provided contacts. |
| Timeliness of lab result | Median number of days from specimen collection to upload into the T2 Salesforce instance (when they would appear in the call queues) among confirmed positive cases. |
| Timeliness of case notification | Median number of days from Maven upload into the T2 Salesforce instance (when they would appear in the call queues) to first case notification attempt among confirmed positive cases. |

eTable 2. Contact Intake Cascade Definitions

| **Metrics** | **Definition for contacts** |
| --- | --- |
| Total Contacts | Contacts were defined as those who were less than 6 feet away from the confirmed or presumed positive case for a total of 15 or more minutes over a 24-hour period during the case’s infectious period (2 days before symptoms for symptomatic case, 2 days before positive molecular or antigen test for asymptomatic cases).  Contacts are found through:   - interviews with confirmed cases who provide their contacts to Trace staff - Bulk uploads from facilities like schools (such as the classmates of a student who is a confirmed case).   All contacts collected through case interviews were included, regardless of vaccination status. Certain school-based contacts were excluded based on CDC criteria. School-based contacts were only included if they are students within 3 ft of a case (or 3-6 ft without a face covering, or staff within 6 ft of a case). Vaccinated, asymptomatic school contacts were not included in total contacts, but were included in other bulk uploaded facilities or through contact collection via case interviews.  Contacts were de-duplicated during contact investigator workflows based on first/last name, address, and phone number, but the time period for deduplication was not known. |
| Attempted | Contacts that were attempted were defined as those where the T2 tracer made at least 1 telephone attempt, including all call dispositions of “busy/no answer”, “incorrect or inactive number”, “left voicemail”, “three failed attempts”, “unable to locate – final”, “call back requested”, “call dropped”, “call back scheduled”, “duplicate/already completed”, “emergency”, “in progress”, “language barrier”, “refused, call completed”, “incapable of responding/no proxy”, “out of jurisdiction”, “potentially deceased”, “referred to NYC Health Department Congregate Settings Team”, “refused – reports vaccination”, and “vaccine immune”.  The proportion of contacts attempted was calculated at the number attempted divided by the total contacts. Attrition from total contacts to attempted was calculated as the percent difference from total contacts to contacts attempted. |
| Reached | Contacts that were reached were defined as those whom the T2 tracer reached, with any call disposition of “call back requested”, “call dropped”, “call back scheduled”, “duplicate/already completed”, “emergency”, “in progress”, “language barrier”, “refused, call completed”, “incapable of responding/no proxy”, “out of jurisdiction”, “potentially deceased”, “referred to NYC Health Department Congregate Settings Team”, “refused – reports vaccination”, and “vaccine immune”.  The proportion of contacts reached was calculated at the number reached divided by the total contacts. The drop-off from attempts to reached was calculated as the percent difference from contacts attempted to contacts reached. |
| Completed | Contacts that were completed were defined as those who completed the intake interview, with call dispositions “call completed,” “incapable of responding/no proxy,” “out of jurisdiction,” “potentially deceased,” “referred to NYC DOHMH Congregate Settings Team,” “refused - reports vaccination,” and “vaccine immune”.  The proportion of contacts completed was calculated at the number completed divided by the total contacts. Attrition from reached to completed was calculated as the percent difference from contacts reached to contacts completed. |
| Eligible monitored | Contacts that were eligible for monitoring were defined as those with completed intake who had >0 days of monitoring left.  The monitoring period was originally specified as 14 days but was changed to 10 days in December 2020.  The proportion of contacts eligible for monitoring was calculated at the number eligible divided by the total contacts. Attrition from completed to eligible was calculated as the percent difference from completed to eligible for monitoring. |
| Success monitored | Contacts that were successfully monitored were defined as those with either at least 1 complete monitoring interaction with ≤3 monitoring days or at least 2 complete monitoring interactions with >4 monitoring days.    The proportion of contacts successfully monitored was calculated as the number successfully monitored divided by the total eligible for monitoring. Attrition from eligible to successful monitoring was calculated as the percent difference from eligible to successfully monitored. |
| Timeliness of contact notification | Median number of days from contact elicitation to first contact notification attempt. For non-bulk uploads, contact elicitation happened during case investigation interviews. |

eFigure 1. Case Investigation Cascade by Age Group, May 31, 2020–January 1, 2022


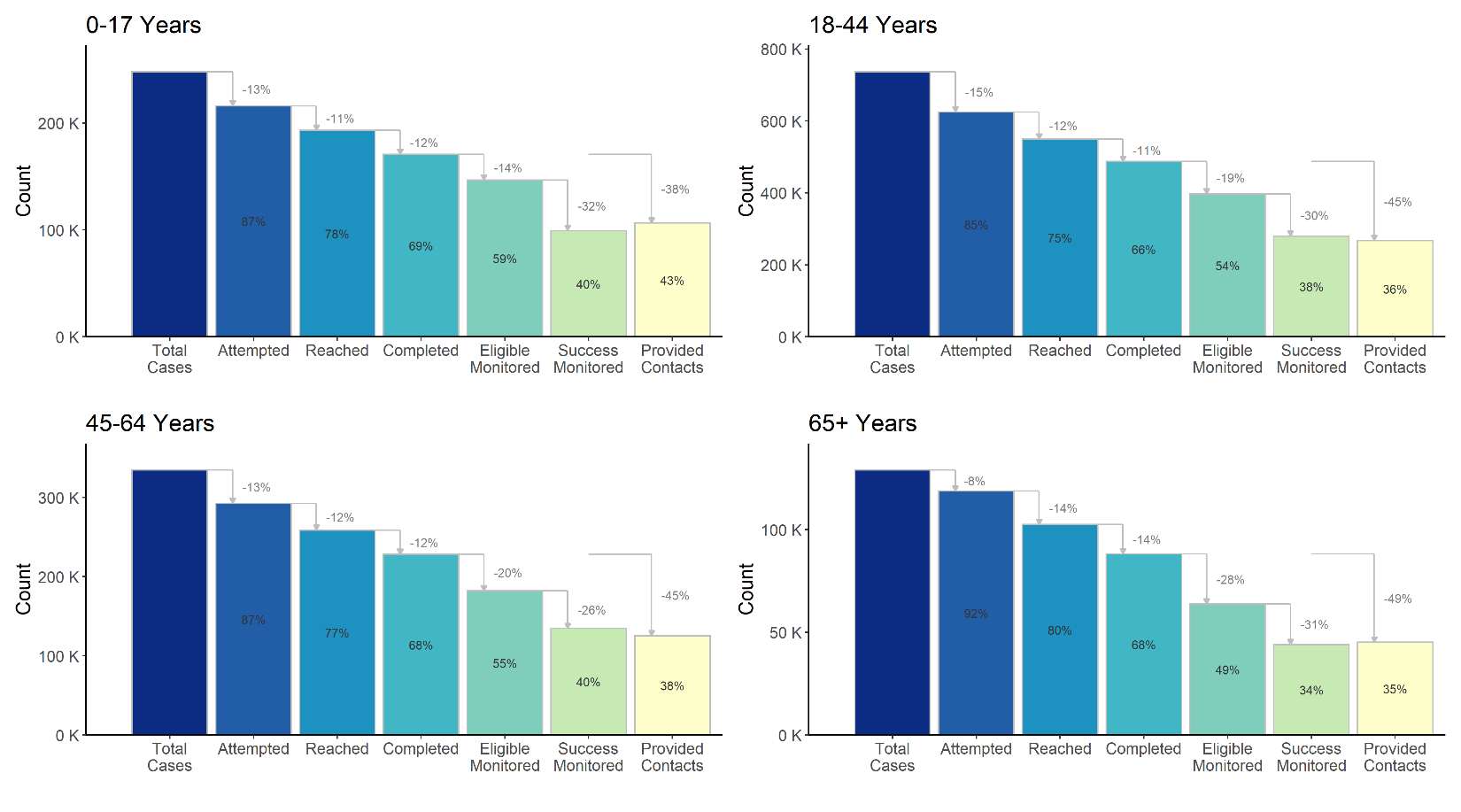


*Percentages outside the bars represent the percent decrease from the reference group displayed by the arrow; percentages inside the bars represent the percent of total cases that reached the cascade category.

eFigure 2. Case Investigation Cascade by Gender, May 31, 2020-January 1, 2022


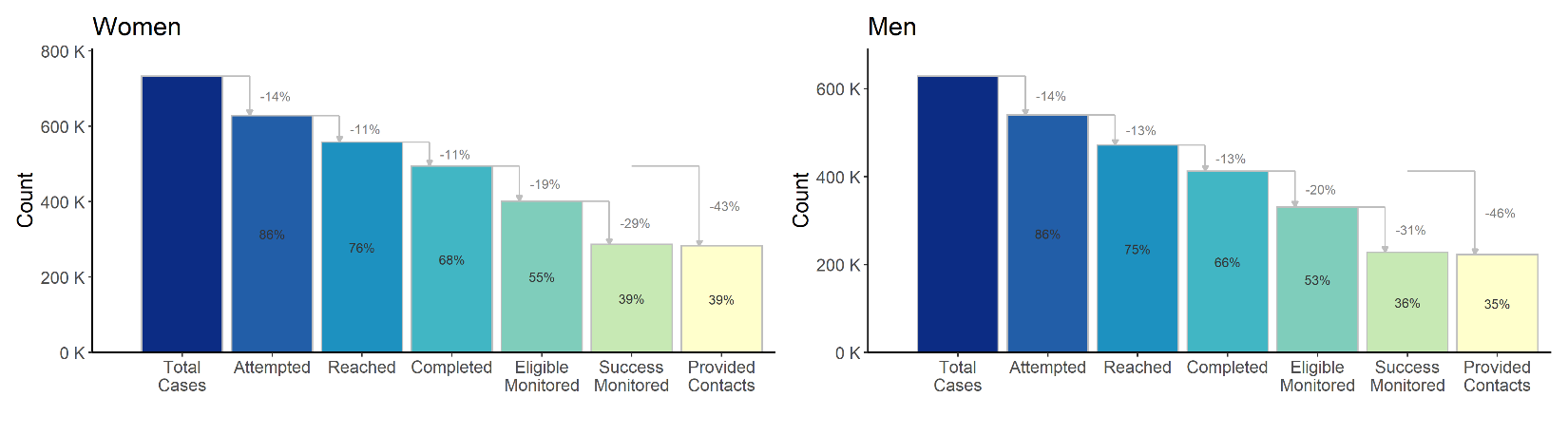
*Percentages outside the bars represent the percent decrease from the reference group displayed by the arrow; percentages inside the bars represent the percent of total cases that reached the cascade category.

eFigure 3. Percent of Cases (Panel A) and Contacts (Panel B) with Completed Intake by ZCTA, May 31, 2020-January 1, 2022

**
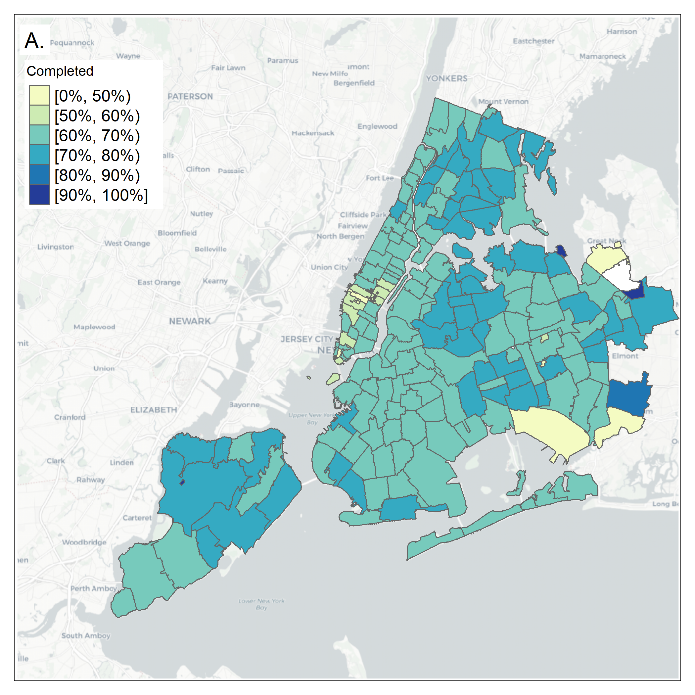

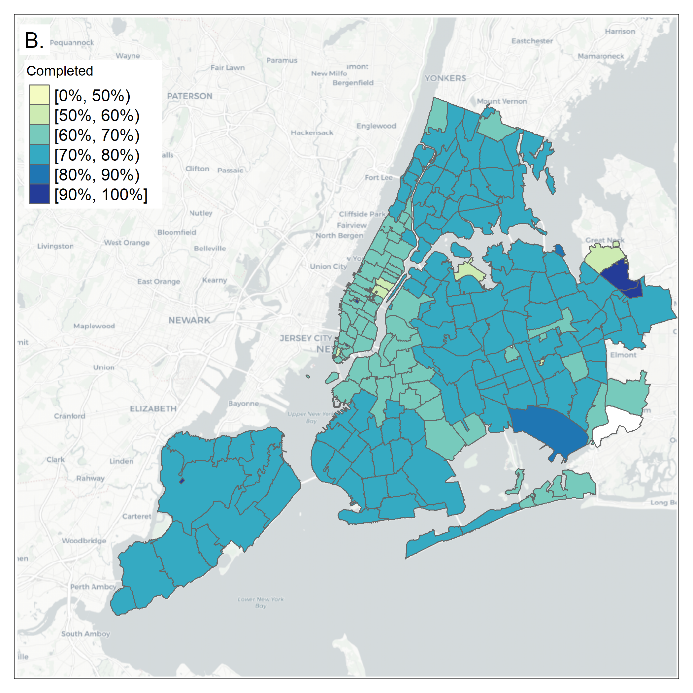
**

eFigure 4. Ratio of Total Cases to Total Contacts by Week, May 31, 2020-January 1, 2022

**
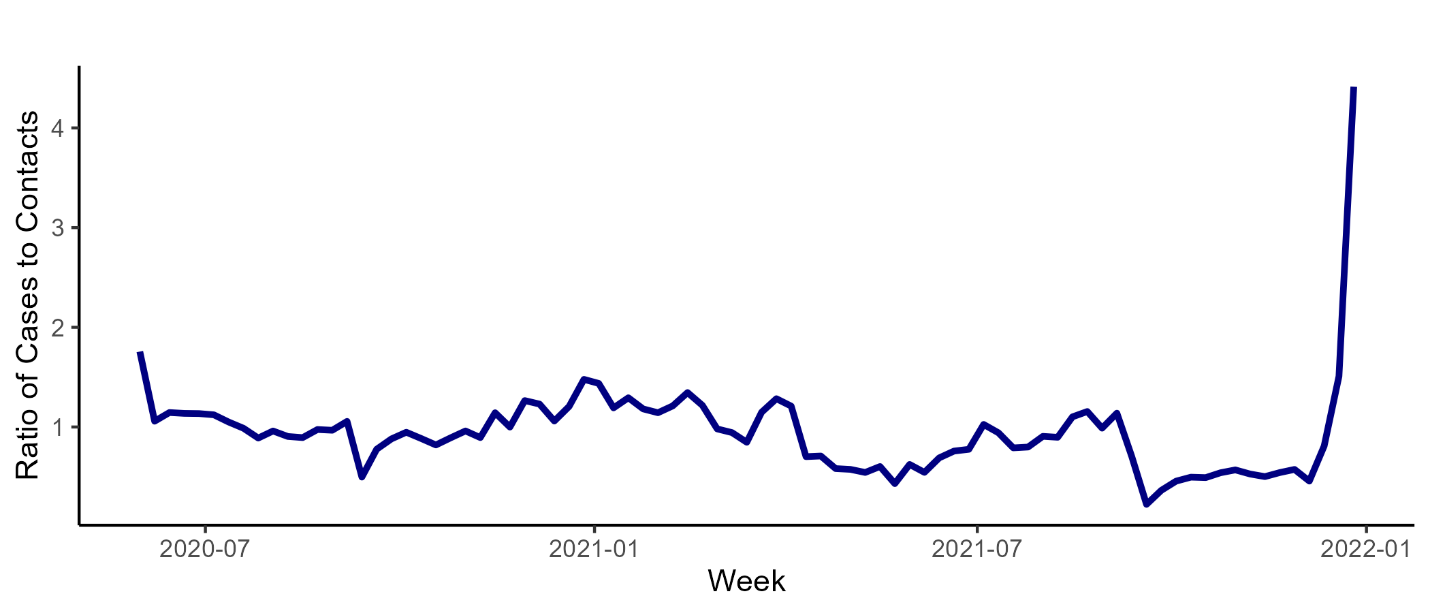
**

eTable 3: Numbers and Proportions of Clients Along the Case Investigation Cascade

| ***Cascade step*** | ***Number*** | ***% of total*** | ***% of prior step*** | ***% lost from prior step*** |
| --- | --- | --- | --- | --- |
| *Total cases* | 1449844 | 100% | - | - |
| *Attempted tracing* | 1253390 | 86.4% | 86.4% | -13.6% |
| *Reached* | 1105221 | 76.2% | 88.2% | -11.8% |
| *Completed tracing* | 975326 | 67.3% | 88.2% | -11.8% |
| *Eligible for monitoring* | 790051 | 54.5% | 81.0% | -19.0% |
| *Successfully monitored* | 557180 | 38.4% | 70.5% | -29.5% |
| *Provided contacts** | 544520 | 37.6% | 55.8% | -44.2% |
| *Prior step is considered to be those who completed tracing. | | | | |

eTable 4: Numbers and Proportions of Clients Along the Contact Intake Cascade

| ***Cascade step*** | ***Number*** | ***% of total*** | ***% of prior step*** | ***% lost from prior step*** |
| --- | --- | --- | --- | --- |
| *Total cases* | 1375455 | 100% | - | - |
| *Attempted tracing* | 1125347 | 82% | 81.8% | -18.2% |
| *Reached* | 817206 | 59% | 72.6% | -27.4% |
| *Completed tracing* | 677349 | 49% | 82.9% | -17.1% |
| *Eligible for monitoring* | 511906 | 37% | 75.6% | -24.4% |
| *Successfully monitored* | 364570 | 27% | 71.2% | -28.8% |
